# Dimensional Model for Estimating Factors influencing Childhood Obesity: Path Analysis Based Modeling

**DOI:** 10.1155/2014/512148

**Published:** 2014-07-07

**Authors:** Maryam Kheirollahpour, Shamarina Shohaimi

**Affiliations:** The Department of Biology, Faculty of Science, University Putra Malaysia, 43400 Serdang, Selangor, Malaysia

## Abstract

The main objective of this study is to identify and develop a comprehensive model which estimates and evaluates the overall relations among the factors that lead to weight gain in children by using structural equation modeling. The proposed models in this study explore the connection among the socioeconomic status of the family, parental feeding practice, and physical activity. Six structural models were tested to identify the direct and indirect relationship between the socioeconomic status and parental feeding practice general level of physical activity, and weight status of children. Finally, a comprehensive model was devised to show how these factors relate to each other as well as to the body mass index (BMI) of the children simultaneously. Concerning the methodology of the current study, confirmatory factor analysis (CFA) was applied to reveal the hidden (secondary) effect of socioeconomic factors on feeding practice and ultimately on the weight status of the children and also to determine the degree of model fit. The comprehensive structural model tested in this study suggested that there are significant direct and indirect relationships among variables of interest. Moreover, the results suggest that parental feeding practice and physical activity are mediators in the structural model.

## 1. Introduction

Structural equation modeling is a powerful statistical tool that combines factor analysis and mathematical modeling to test the hypotheses that consisted of interacting variables and pathways with reference to substantive theory. This method is widely employed where indicator variables, such as parental feeding practices cannot be readily measured and have to be derived from questionnaires [1, 2].

Obesity has clearly become one of the most important public health problems of the late twentieth century and is now being recognized as a serious threat to society due to its increasing prevalence [3, 4]. As a result, preventive efforts must focus on the population as a whole. Childhood obesity is an unhealthy body condition that is mostly caused by gaining weight more than usual. Studies have shown that socioeconomic status of parents, parental feeding practice, and children's physical activity are the most important factors affecting children's obesity [5–11]. Hence, the estimation and measurement of these factors have constituted an important topic for studies in recent decades. Previously, different regression models have been applied to estimate the factors that affect children's weight status [12–17]. In studying the regression models, it is apparent that they do not present a comprehensive model to assess all the factors concerning children's obesity; therefore, they cannot be applied for comprehensive evaluation. Another problem with regression model is the presence of multicollinearity. Although multicollinearity does not decrease the predictive power of the regression model, it affects individual predictors. In other words, although a multiple regression model with correlated predictors may indicate that the entire model is good fitted, it may not provide valid results about any individual predictor. Besides, the new model presents the overall effects of both the observed and the unobserved factors that lead to weight gain in children. In addition, the proposed model in this study seeks to estimate the dependent variables (parental feeding practice and physical activity) based on definitions of the latent variables.

Consequently, one comprehensive model could provide an overall assessment of the constructs by using a combination of the four indicators (socioeconomic status of parents, parental feeding practice, physical activity, and weight status of children).

## 2. Material and Methods

This is a cross-sectional study that was conducted in the state of Selangor and Federal Territory of Kuala Lumpur. A total of 379 students, 7–9 years old, participated. Most studies achieved a substantial link between the variables of interest by using regression models [12–14, 16]. Structural equation modeling (SEM) is a suitable method for testing the relationships among variables [18]. SEM is capable of checking and examining a complete model generating wellness of fit statistics and assessing the overall fit of the complete model at the same time [19].

The current study examined six important relationships among variables. First, the direct relation of socioeconomic status and children weight status were analyzed to investigate whether income and educational level of parents relate to children's gain of weight. Second, the direct relation of socioeconomic status and parental feeding practice, as well as socioeconomic status and physical activity level of children were evaluated to understand whether socioeconomic status was associated with parental feeding practice and children physical activity. Third, the direct relation of physical activity and weight status of children was assessed to examine that how physical activity level of the children related to their weight status. Fourth, the direct relation of parental feeding practice and child weight status was examined to understand whether feeding practice is related to weight status of children. Finally, the indirect relation of socioeconomic status and children weight status through parental feeding practice and physical activity were analyzed to investigate whether the parental feeding practice and physical activity serve as mediators between socioeconomic status and children weight status.

According to Gerbing and Anderson [20], the SEM procedure has three main parts: confirmatory factor analysis (CFA), producing the measurement model, and conducting the structural model. The confirmatory factor analysis is used to test the validity and suitability of the indicators for each construct [21]. The outcome from this procedure is the goodness of fit values for each construct. The commonly used fit indices in the literature include the related Chi-square statistics (CMIN), comparative fit index (CFI), Tukey-Lewis index (TLI), and root mean square error of approximation (RMSEA). According to Garson [22] CFI and TLI measures equal to or greater than 0.9 signify good fit indices. Also, RMSEA less than 0.05 displays the most acceptable fit index [23], while CMIN must be less than 0.5 to show a good fit index. For evaluation of the models (structural model and confirmatory factor analysis), the Chi-square statistics are expected to be nonsignificant and at least four indices must be significant. Besides complete structural relationships, indirect and direct relations among exogenous variable and endogenous variable were completely specified by confirmatory factor analysis. Finally comprehensive model should be tested for the presence of mediators and moderator in model.

Some fundamental terms and concepts which have applied in this study such as Indicators (apparent or reference variable), are known as observed variable. Unobserved (latent) variable/construct is an unobserved (latent) variable that is measurable by its index. Observed indicators are graphically showed by squares and unobservable (latent) indicators are represented by oval. Multistage stratified sampling was used in this study due to the ethnic distribution of the population in Malaysia. Comprehensive Feeding Practices Questionnaire (CFPQ) that includes parent's socioeconomic status questionnaire was applied. The Physical Activity Questionnaire (PAQ-C) was used to assess general levels of physical activity of the children.

Six important relationships were investigated in the form of a current study of six hypotheses. The following hypotheses were examined.

1. There is significant association between socioeconomic status and children weight status.
2. There is significant association between socioeconomic status and parental feeding practice.
3. There is significant association between socioeconomic status and physical activity level of children.
4. There is significant association between physical activity and weight status of children
5. There is significant association between parental feeding practice and child weight status.
6. Finally, there is significant association between socioeconomic status and children weight status through parental feeding practice and physical activity in one comprehensive model.

There were five constructs in the current study; three of them were latent variables that consisted of socioeconomic status (exogenous independent variable), feeding practice, and physical activity (endogenous variables) and two of them were measurement that include weight status (BMI) of children (dependent) and weight status (BMI) of mother. Usually identified structural model is not perfectly fitted to the data. Therefore, the degree of model fitness must be measured. The Chi-square, CFI, RMSEA, and TLI were some of the most common indices which were available to evaluate the model fit. In the first step, each latent variable was checked to determine the degree of model fit, which explained the variances and standardized residual for the measurement variables and the adequacy of the factor loadings.

## 3. Result and Discussion

**Figure 1:**
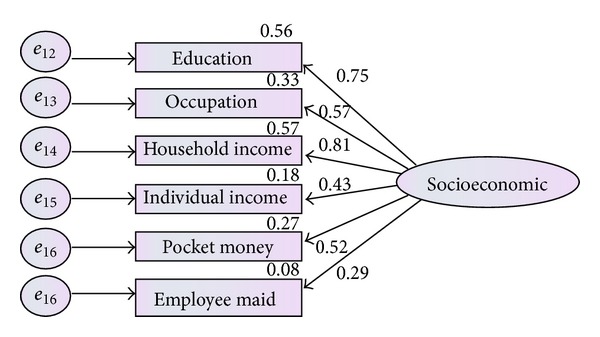
Measurement model for socioeconomic status before factor loading.

To provide evidence for construct validity of latent variables (socioeconomic status, parental feeding practice and physical activity), three separate CFA models were assessed. Only valuable indicators of each latent variable would be maintained in model by utilizing factor loading. The indicators of socioeconomic status were shown by measurement model in Figure 1. Each number on path represented the factor loading between socioeconomic factor (latent construct) and items.

**Figure 2:**
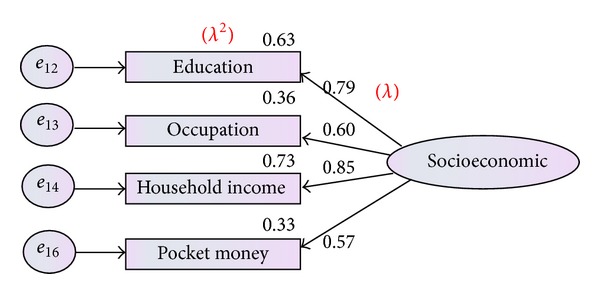
Modified measurement model for socioeconomic factors.

For the first construct (socioeconomic status) as presented in Table 1, the CFA model did not indicate the adequate fit to the given data in terms of (*χ* ^2^(13,379) = 28.417, *P* value <0.05, TLI = 0.893 and RMSEA = 0.117), but the other indices were acceptable. According to Hair et al. [24] and Byrne [23], standardized factor loading must meet these three criteria. First, each factor loading must be more than 0.5, also none of factor loadings must be neither negative nor more than one [24]. As presented in Figure 1, factor loading of individual income and employee maid were less than 0.5; therefore, these two items must be eliminated from the measurement model. Therefore, modified model for socioeconomic status would be presented as Figure 2.

Table 2 illustrates that all the indices meet the criteria after factor loading; (*χ* ^2^(13,379) = 16.724, *P* value <0.05, CFI = 0.930, TLI = 0.943, CMIN = 1.04, GFI = 0.9, and RMSEA = 0.014). Therefore, socioeconomic status of family could be defined by four indicators as presented in Figure 2, including education level, occupation level, household income, and amount of pocket money given to children.

**Figure 3:**
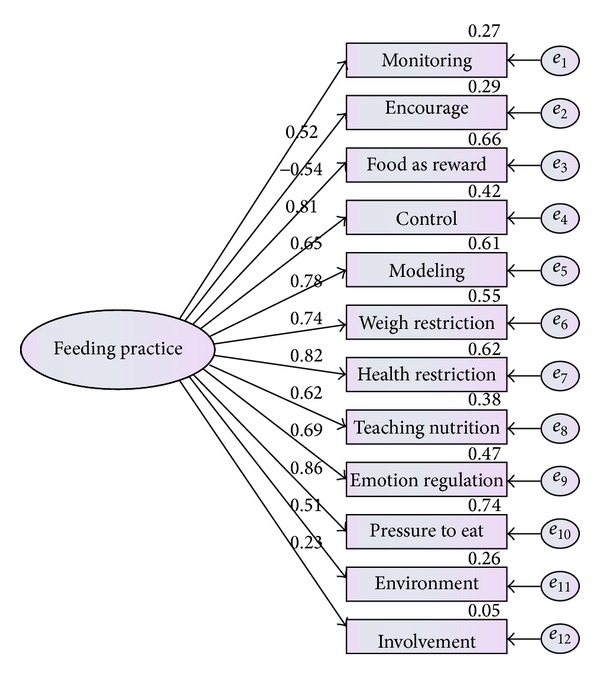
Measurement model for parental feeding practice.

Similar to socioeconomic status, parental feeding practice was described by twelve items. These twelve items were examined to check the adequacy of the measure of each item. As result shown in Table 3 and Figure 3, the measurement model did not indicate the adequate fit to the given data in terms of (*χ* ^2^(11,379) = 46.424, *P* value <0.05, CMIN = 12.640, TLI = 0.887, and RMSEA = 0.265), but the other indices were acceptable.

**Figure 4:**
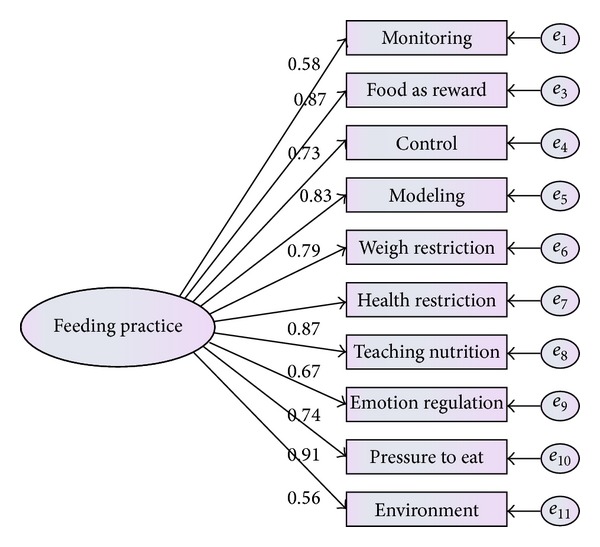
Modified measurement model for parental feeding practice.

As can be seen from Figure 3, involvement and encourage with 0.23 and −0.54 coefficients did not meet the criteria, so they should be removed from the model. Result presented that modified model is fitted well. The model after eliminating these factors is presented by Figure 4.

The result obtained by confirmatory factor analysis in Table 4 and Figure 4 showed that the measurement model was well fitted to the current data.

**Figure 5:**
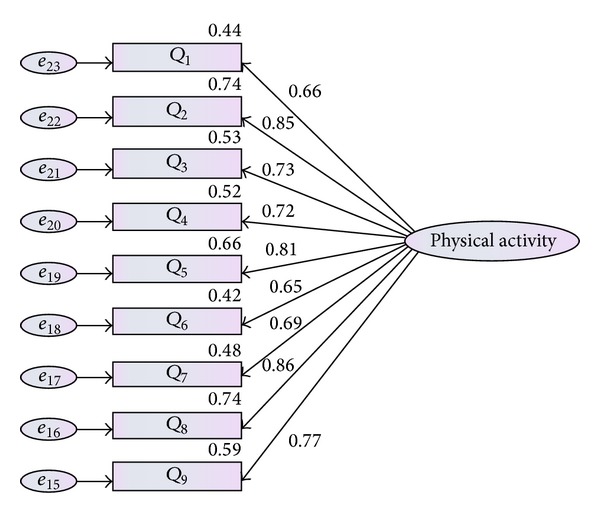
Measurement model for physical activity.

The last latent variable which was taken under consideration was physical activity. Physical activity was defined by nine items (nine questions of physical activity questionnaire). As result shown in Table 5 and Figure 5, the CFA model indicated the adequate fit to the given data in terms of (*χ* ^2^(8,379) = 13.524, *P* value <0.05, CMIN = 1.940, TLI = 0.977, and RMSEA = 0.051).

## 4. Measurement Model

**Figure 6:**
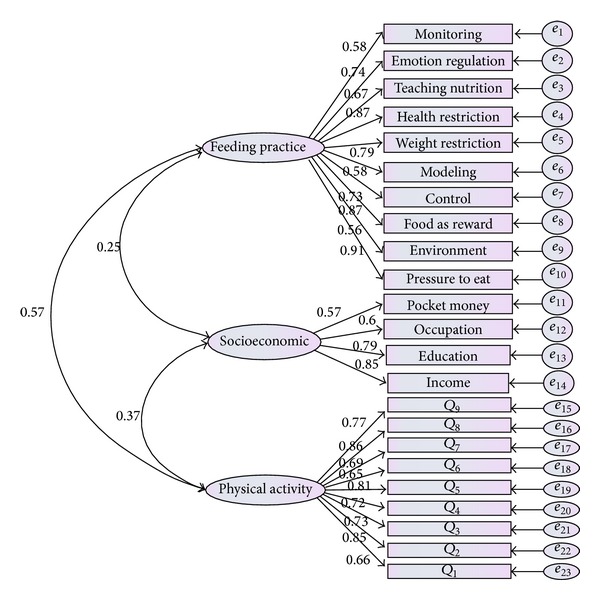
Measurement model in same line (without exogenous or endogenous variables).

Existence of multicollinearity was a serious threat to the SEM. Usually low discriminant validity of the factors will cause the multicollinearity. In order to test the discriminant validity of the factors, measurement model must be checked. The full model without considering the exogenous or endogenous variable would be presented. Regarding this, all latent variables were considered in one level. In Figure 6, curve lines showed the covariance between latent variables. As illustrated in Table 6, all the indices meet the criteria; (*χ* ^2^(22,379) = 136.224, *P* value <0.05, CMIN = 3.640, CFI = 0.915, TLI = 0.955, GFI = 0.914, and RMSEA = 0.023). The full measurement model of this study was fitted to data well. In addition, Figure 6 showed that none of factor loading coefficients was less than 0.5. Also the covariance between the constructs that was shown in Figure 6 illustrated a strong relation between these latent variables (parental feeding practice, physical activity, and socioeconomic status).

According to Kline [25], the high correlation between two latent constructs, greater than 0.85, shows the multicollinearity [25]. Based on the result of Figure 6, correlation among the latent constructs was not greater than 0.85 and consequently the multicollinearity does not exist.

## 5. Structural Model

Structural model was used to identify the assumed relation between the constructs (endogenous or exogenous) which was linked to the hypothesized model's constructs.

*Hypothesis 1.* There is significant association between socioeconomic status and parental feeding practice.

The first hypothesis was tested to examine whether socioeconomic status related to parental feeding practice. The model provided perfect fit to the given data (*χ* ^2^(13,379) = 13.714, *P* value <0.05, CFI = 0.922, TLI = 0.933, CMIN = 1.14, GFI = 0.93, and RMSEA = 0.011).

**Figure 7:**
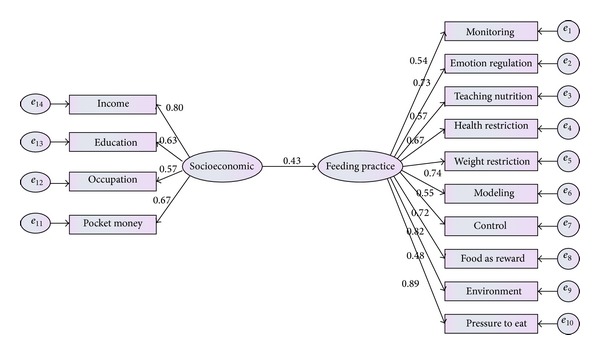
Standardized path coefficients and residual variance in the first model.

As presented in Figure 7, socioeconomic status had positive significant relation with parental feeding practice (*β* = 0.43, *P* < 0.05). Parental feeding practices were more practiced in family with higher socioeconomic status. 

*Hypothesis 2.* There is significant association between socioeconomic status and physical activity.

**Figure 8:**
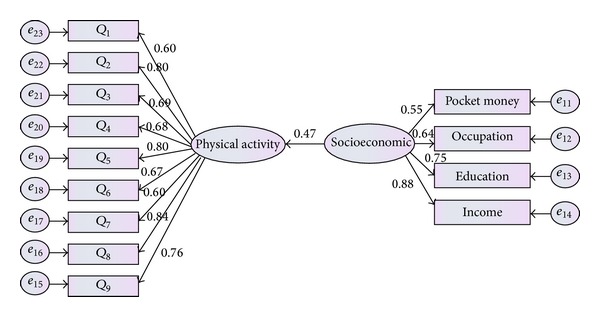
Standardized path coefficients and residual variance in the second model.

The second hypothesis was tested to investigate whether socioeconomic status directly related to physical activity. The model presented in Figure 8 provided the reasonable fit to the given data (*χ* ^2^(12,379) = 13.714, *P* value <0.05, CFI = 0.935, TLI = 0.901, CMIN = 1.03, GFI = 0.99, and RMSEA = 0.041).

*Hypothesis 3.* There is significant association between socioeconomic status and weight status of children (BMI).

The third hypothesis was analyzed to investigate the relation between socioeconomic status and weight status of children. The model provided the reasonable fit to the given data (*χ* ^2^(4,379) = 24.456, *P* value <0.05, CFI = 0.930, TLI = 0.90, CMIN = 1.08, GFI = 0.96, and RMSEA = 0.037).

**Figure 9:**
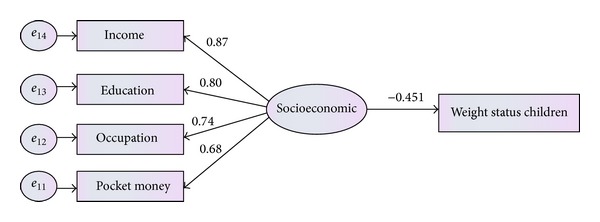
Standardized path coefficients and residual variance in the third model.

Result of Figure 9 showed that socioeconomic status had negative significant relationship with children's weight status with coefficient (*β* = −0.451, *P* < 0.05). It could be said that children of higher socioeconomic status were more likely to have lower BMI.

*Hypothesis 4.* There is significant association between parental feeding practice and weight status of children (BMI).

The fourth hypothesis was examined to test whether parental feeding practice was related directly to the weight status of children (BMI). The model provided a good fit to the data (*χ* ^2^(9,379) = 26.332, *P* value <0.05, CFI = 0.97, TLI = 0.95, CMIN = 1.056, GFI = 0.90, and RMSEA = 0.041).

**Figure 10:**
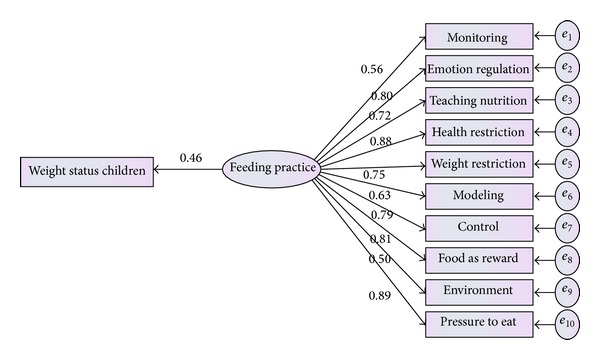
Standardized path coefficients and residual variance in the fourth model.

As presented in Figure 10, parental feeding practices (*β* = 0.46, *P* < 0.05) were directly related to weight status of children. Parents with higher consideration about feeding practice were more likely to have children with lower BMI.

*Hypothesis 5.* There is significant association between physical activity and weight status of children.

The fifth hypothesis was to understand the direct relation between physical activity and weight status of children. As result shown (*χ* ^2^(8,379) = 31.112, *P* value <0.05, CFI = 0.96, TLI = 0.987, CMIN = 1.913, GFI = 0.92, and RMSEA = 0.038) the model provided an adequate fit to the data.

**Figure 11:**
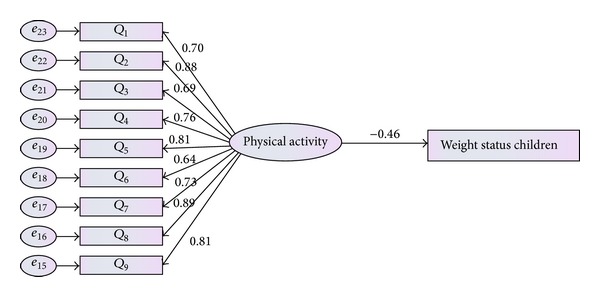
Standardized path coefficients and residual variance in the fifth model.

Result of Figure 11 indicated that there was a significant negative relationship between physical activity and weight status of children (*β* = −0.46, *P* < 0.05). Clearly children who were more involved in physical activity were more likely to have low BMI.

*Hypothesis 6.* Full structural model was able to examine the entire relationship among variables.

The final hypothesis was tested to examine the full relationship among constructs. Testing the previous five hypotheses provided strong support to investigate the entire relationship in full model. This test was comparable to testing hypothesis of regression in multiple regression models. Results in Table 7 showed that related that the Chi-square is equal to 1.064 and at least four of the indices have met the criteria. (*χ* ^2^(8,379) = 35.672, *P* value <0.05, CFI = 0.966, TLI = 0.859, CMIN = 1.064, GFI = 0.966, and RMSEA = 0.051). Model provided an adequate fit to the data.

**Figure 12:**
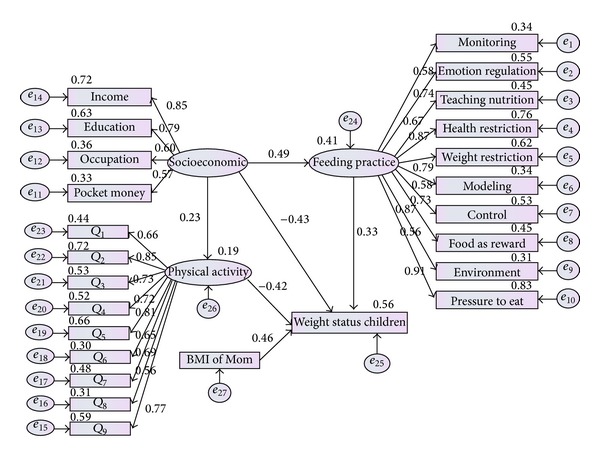
Comprehensive structural model with regression weight and covariance coefficients.

Result of Figure 12 showed that there were significant relationship between socioeconomic factor and feeding practice (*β* = 0.49, *α* < 0.05) and significant relationship between socioeconomic status and children weight status (*β* = −0.43, *α* < 0.05). Moreover a significant positive relation was seen between socioeconomic status and physical activity (*β* = 0.23, *α* < 0.05) as well as feeding practice and weight status of children (*β* = 0.33, *α* < 0.05). Finally, a significant negative relation between physical activity and weight status of children was investigated (*β* = −0.42, *α* < 0.05). Therefore, it seems that this model was significantly fitted to data. Result of full structural model with regression path coefficient was also presented in Table 8. According to Figure 12, 41% of variance of feeding practice would be explained by socioeconomic factors, while 56% of variance of weight status of children would be identified by socioeconomic status, physical activity, and feeding practice and only 19% of variance of physical activity would be explained by SES.

## 6. Direct, Indirect, and Total Association

In order to test the direct and indirect relationship in the model, two paths must be considered. The first one indicated the relation between the socioeconomic status and weight status through feeding practice and the second one showed the relation between socioeconomic and weight status according to physical activity. Both indirect and direct relations were examined by SEM and results were presented in Tables 9 and 10.

Result of Tables 9 and 10 indicated that socioeconomic status directly predicted physical activity (*β* = 0.23, *α* < 0.05) and parental feeding practice (*β* = 0.49, *α* < 0.05). Interestingly, physical activity was negatively associated with weight status of children (*β* = −0.43, *α* < 0.05). Result in Table 9 suggested that socioeconomic status had a significant indirect relation with weight status of children (*β* = 0.10, *α* < 0.05) through its effect on physical activity. On the other hand, parental feeding practice positively associated with weight status of children (*β* = 0.33, *α* < 0.05). Result of Table 10 indicated that socioeconomic status had a significant indirect relationship with children weight status (*β* = 0.16, *α* < 0.05) through parental feeding practice.

## 7. Mediation Model

**Figure 13:**
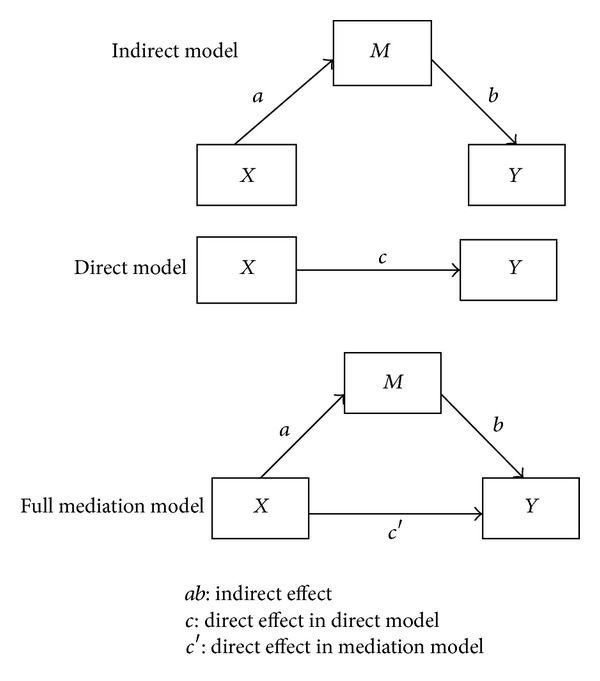
Indirect, direct, and full mediation model [27].

According to Hair et al., a mediation effect would be created when a third construct intervened between two other related constructs [26, 27]. According to Kline [28], in order to examine the mediation model, the full mediation model must be tested versus indirect model. The presence of mediators would be accepted if the full mediation model was better fitted than the indirect model. In this regard three different models were created with names of direct model, indirect model, and full mediation model as in Figure 13.

Result shown in Table 11 represented the mediation model is significantly better fitted to the data than indirect model while feeding practice and physical activity were added to the model.

Result of Table 12 showed that all relations in both models (direct and full mediation) were found significant, then it can be concluded that this model was partially mediated by feeding practice and physical activity [28].

## 8. Discussion and Conclusion

The main purpose of this study was to examine the direct and indirect relations among the socioeconomic status, parental feeding practice, physical activity, and weight status of children by testing the hypothesized structural model based on path analysis. Furthermore, the current study attempted to provide a valid and reliable model to measure the factors related to obesity in children. In the present study, confirmatory factor analysis suggested that each item in three latent variables (socioeconomic status, parental feeding practice, and physical activity) had acceptable factor loadings ranging from 0.53 to 0.91 on their latent variables. Regardless of the validity of the comprehensive model, which was able to estimate the influential indicators of childhood obesity, associations between parental feeding practice and childhood obesity were identified using this comprehensive model, as well as socioeconomic factors and physical activity. In addition, it could be said that this model was a multimediation model in that two mediation variables were recognized in the model. The characteristics of the sample of this study were defined as 252 Malays, 91 Chinese, and 36 Indians by considering the ratio of the distribution of the population in Malaysia. All respondents were individuals who aged 7 to 9 years old.

Recent studies in childhood overweight and obesity [12, 29–32] provide strong evidence for the association between the socioeconomic status and weight status of children. Similarly, the finding of the present study indicated that socioeconomic factors were strongly associated with the weight status of children.

Another relationship that was investigated by this study was the relation between the socioeconomic status and parental feeding practices. The results of the current study indicated that socioeconomic status is strongly related to feeding practices. In other words, parents with higher income or educational level were more likely to teach their children about nutrition, modeling them, encouraging them, or creating a healthy environment at home. These findings were supported by many studies [33, 34].

Among the items of feeding practice, only two items (pressure to eat and restriction) were considered by many studies [35–37]. Although the model fitted the data well and all the linked hypotheses were found to be significant, there was less evidence of previous studies to support the model inasmuch as the application of structural equation modeling in health and nutrition studies is relatively new.

The relationship between the socioeconomic status and involvement in physical activity was another important relation that was examined by the current investigation. The results indicated that there was a powerful relation between the socioeconomic status of the parents and involvement in physical activity. The evidence indicated that parents of high social level (income and education) were more knowledgeable about the benefits of physical activity, and also it could be said that higher income would enable parents and children to easily reach physical facilities. These findings were supported by similar research concerning physical activities [38]. The brief explanations of the significant relationship among the variables in the model are presented in Table 13.

Compared to similar research, physical activity and whole subscales of feeding practice have recently been added to the new model. Therefore, this model could be more extensive and could cover more useful information about the factors that lead to overweight and obesity in children. In addition, the model was fitted to this sample of data. All the hypotheses were adequately supported by the data.

It was proven that socioeconomic status, with 0.43 of regression weight, was significantly related with the weight status of children and that parental feeding practice was also significantly associated with the weight status of children. Moreover, physical activity was found to be an influential factor in relation to the weight status of children. The BMI of the mother had a significantly positive relation with the BMI of their children. It seems that mothers with higher BMI were more likely to have heavier children.

Even though the fit indices of the structural model suggested an adequate fit to the data, some path coefficients provided contradictory results to the previous models suggested by other studies. Most specifically, the path coefficient between parental feeding practice and children's weight status indicated a positive association. This association was reported negatively by other studies [39–41].

A larger amount of the shared variances of the variables in the model were identified through the hypothesized model due to the strong relation between the constructs. In other words, from the results, 56% of changes in the weight status of children could be explained by the socioeconomic status and feeding practice as well as physical activity. A good fitting structural model to the observed data indicates that the model was consistent with the relationships within the observed data.

One of the main purposes of this study was to investigate the dimensional model to evaluate the direct and indirect relationships between the constructs. Although the regression models in previous studies were able to define and estimate the relation among the constructs, they were not able to identify the direct and indirect relations between the variables of interest. The findings of this study suggested that, regardless of the direct relations between the socioeconomic status and weight status of children, there was an indirect significant relationship between the socioeconomic status and weight status of children through parental feeding practice. Parents with higher socioeconomic status (higher income and education level) were more likely to have children with lower BMI. Moreover, parents with higher education and income level were more able to use several types of feeding practice. Therefore, their children were more likely to have normal weight. It could be said that the socioeconomic status of parents (income level and education level mostly), in adding to having direct relations to a child's weight status, could influence the weight status of children by affecting the parents through feeding practice. Similarly, some researchers indicated the mediation role of parental feeding practice in the model [35, 42].

Furthermore, physical activity is strongly related to a child's BMI. Although the direct impact of physical activity on a child's BMI was accepted and examined by many studies [43–46], it was also considered as a mediator between the relationship of socioeconomic status and children's weight status. Similarly, the results of the current investigation emphasized that the socioeconomic status could be indirectly related to the weight status of children through the impact of physical activity. As mentioned before, while the socioeconomic status of the parents was directly related to the weight status of the children, it could also be related to the weight status of the children through its influence on the physical activity level of the children. In other words, children of higher socioeconomic class were more able to access physical activity facilities.

The model presented by this study was recognized as partial mediation, due to the mixed variables in the model, which consists of three latent variables (feeding practice, socioeconomic status, and physical activity) and two manifest variables (BMI of mother and weight status of children). The findings from the full structural model suggested that parents with a high social level and who utilize more feeding practices might have children with a lower BMI. In other words, feeding practice was identified as a mediator in relation to socioeconomic status and weight status of children.

Furthermore, the full structural model provides evidence of a significant increase in the strength of the path coefficient between the socioeconomic status and the weight status of children when the components of physical activity were included in the model.

This means that social level with maternal feeding practice and physical activity could have a stronger relation with children's weight status. The results obtained by SEM completely correspond with the results obtained by other studies [47–49]. It could be said that the model was recognized as a multimediation model due to the two mediators identified in this model.

There are some limitations in this study that should be accounted. The first limitation of the present study was the lack of evidence to support a link between the intake of calories of the children and the expenditure of energy for physical activity. Another issue that has to be taken into consideration was the genetic roots of being overweight and obese. Genetics are among the important factors that cause obesity [50] and could be added to the model. Although the clinical cause and effect were not considered in this study, due to the importance of these items, it should be investigated in future experimental studies. Generally, the study was strengthened by applying a sufficient sample size in order to achieve scientifically reliable results by applying SEM. Finally, structural equation modeling was used to analyze the data. Even though several models were tested and the final model was investigated as comprehensive model, using different frameworks, researchers can generate different structural models to examine the relationships between socioeconomic status of family and weight status of children.

## Figures and Tables

**Table 1:** Factor loading for socioeconomic status.

Fit index	Value	Acceptance area	Acceptability
Chi-square fit (*P*-value)	28.417	Less better	−
CMIN/df (related *χ* ^2^)	6.340	<5.0	+
GFI	0.900	>0.9	+
RFI	0.975	>0.9	+
NFI	0.904	>0.9	+
IFI	0.919	>0.9	+
TLI	0.893	>0.9	−
CFI	0.918	>0.9	+
RMSEA	0.117	<0.08	−

**Table 2:** Factor loading for modified model of socioeconomic status.

Fit index	Value	Acceptance area	Acceptability
Chi-square fit (*P*-value)	16.724	Less better	
CMIN/df (related *χ* ^2^)	1.040	<5.0	+
GFI	0.900	>0.9	+
RFI	0.974	>0.9	+
NFI	0.913	>0.9	+
IFI	0.965	>0.9	+
TLI	0.943	>0.9	+
CFI	0.930	>0.9	+
RMSEA	0.014	<0.08	+

**Table 3:** Factor loading for Parental feeding practice.

Fit index	Value	Acceptance area	Acceptability
Chi-square fit (*P*-value)	46.424	Less better	−
CMIN/df (related *χ* ^2^)	12.640	<5.0	−
GFI	0.908	>0.9	+
RFI	0.919	>0.9	+
NFI	0.917	>0.9	+
IFI	0.965	>0.9	+
TLI	0.887	>0.9	−
CFI	0.965	>0.9	+
RMSEA	0.265	<0.08	−

**Table 4:** Modified factor loading for parental feeding practice.

Fit index	Value	Acceptance area	Acceptability
Chi-square fit (*P*-value)	13.524	Less better	+
CMIN/df (related *χ* ^2^)	1.940	<5.0	+
GFI	0.921	>0.9	+
RFI	0.935	>0.9	+
NFI	0.955	>0.9	+
IFI	0.972	>0.9	+
TLI	0.977	>0.9	+
CFI	0.965	>0.9	+
RMSEA	0.051	<0.08	+

**Table 5:** Factor loading for physical activity.

Fit index	Value	Acceptance area	Acceptability
Chi-square fit (*P*-value)	27.773	Less better	+
CMIN/df (related *χ* ^2^)	2.602	<5.0	+
GFI	0.911	>0.9	+
RFI	0.943	>0.9	+
NFI	0.919	>0.9	+
IFI	0.907	>0.9	+
TLI	0.962	>0.9	+
CFI	0.980	>0.9	+
RMSEA	0.043	<0.08	+

**Table 6:** Confirmatory factor analysis for final measurement model.

Fit index	Value	Acceptance area	Acceptability
Chi-square fit (*P*-value)	136.224	Less better	−
CMIN/df (related *χ* ^2^)	3.640	<5.0	+
GFI	0.914	>0.9	+
RFI	0.920	>0.9	+
NFI	0.982	>0.9	+
IFI	0.887	>0.9	+
TLI	0.955	>0.9	+
CFI	0.915	>0.9	+
RMSEA	0.023	<0.08	+

**Table 7:** Confirmatory factor analysis for testing structural model fit.

Fit index	Value	Acceptance area	Acceptability
Chi-square fit (*P*-value)	35.672	Less better	
CMIN/df (related *χ* ^2^)	1.064	<5.0	+
GFI	0.944	>0.9	+
RFI	0.928	>0.9	+
NFI	0.962	>0.9	+
IFI	0.991	>0.9	+
TLI	0.859	>0.9	−
CFI	0.966	>0.9	+
RMSEA	0.051	<0.08	+

**Table 8:** Regression weight for individual path in full structural model.

Casual path	Coefficient	C.R	*P*-value
Socio-economic→feeding practice	0.49	2.045	0.031∗
Socio-economic→weight status children	−0.43	4.310	0.000∗
Socio-economic→physical activity	0.23	5.489	0.000∗
Feeding practice→weight status	0.33	4.276	0.000∗
Physical activity→weight status	−0.42	5.065	0.000∗
BMI of mother→weight status	0.36	3.214	0.000∗

*Significant in *α* level of 0.05.

**Table 9:** Socioeconomic status related to weight status of children (through physical activity).

Model	Variables	*α*-value	*B*

Direct relationship	Socioeconomic→ weight status of children	0.05	−0.43
Indirect relationship	Socioeconomic→ physical activity→ weight status	0.05	0.10

Total effect	Direct + indirect	0.05	0.33

**Table 10:** Socioeconomic status related to weight status of children (through feeding practice).

Model	Variables	*α*-value	*B*

Direct relationship	Socioeconomic→ weight status of children	0.05	0.43
Indirect relationship	Socioeconomic→ feeding practice→ weight status	0.05	0.16

Total effect	Direct + indirect	0.05	0.59

**Table 11:** Comparison between full mediation model and indirect model for mediation role of feeding practice and physical activity.

Model	CMIN	*α*-value	Criteria
Full mediation model (feeding practice)	0.225	0.05	Accepted
Indirect model	0.037	0.05	
Full mediation model (physical activity)	0.107	0.05	Accepted
Indirect model	0.012	0.05	

According to Kline [28] sig *χ* ^2^ > *α*.

**Table 12:** Compression between full mediation model and direct model.

Construct	Beta	*P*-value	
Direct model			
Socioeconomic→ weight status	0.323	0.000	Significant
Full mediation model			
Socioeconomic→ weight status	0.217	0.000	Significant
socioeconomic→ feeding practice	0.321	0.000	Significant
feeding practice→ weight status	0.274	0.000	Significant

**Table 13:** Results of the testing of the hypotheses.

Hypothesis	Supported

H_1_: There is significant relationship between SES and PFP	Yes
H_2_: There is significant relationship between SES and PA	Yes
H_3_: There is significant relationship between SES and the weight status of children	Yes
H_4_: There is significant relationship between PFP and the weight status of children	Yes
H_5_: There is significant relationship between PA and the weight status of children	Yes

SES: socioeconomic status, PFP: parental feeding practice, PA: physical activity.
